# Quantum-SAR Extension of the Spectral-SAR Algorithm. Application to Polyphenolic Anticancer Bioactivity

**DOI:** 10.3390/ijms10031193

**Published:** 2009-03-16

**Authors:** Mihai V. Putz, Ana-Maria Putz, Marius Lazea, Luciana Ienciu, Adrian Chiriac

**Affiliations:** 1 Laboratory of Computational and Structural Physical Chemistry, Chemistry Department, West University of Timişoara, Pestalozzi Street No.16, Timişoara, RO-300115, Romania; E-Mails: lacrama.anamaria@cbg.uvt.ro (M.P.); laz_marius2@yahoo.com (M.L.); achiriac@cbg.uvt.ro (A.C.); 2 “Nicolas Georgescu-Roegen” Forming and Researching Center, 4th, Oituz Str., Timişoara, RO- 300086, Romania; 3 Laboratory of Inorganic Chemistry, Timişoara Institute of Chemistry of Romanian Academy, Av. Mihai Viteazul, No.24, Timişoara RO-300223, Romania; 4 Whatman, Part of GE Healthcare, Inc, 200 Park Avenue Suite 210, Florham Park, NJ 07932-1026, USA; E-Mail: Luciana.ienciu@ge.com

**Keywords:** QSAR, correlation factors, vector norms, spectral paths, flavonoids, EC50

## Abstract

Aiming to assess the role of individual molecular structures in the molecular mechanism of ligand-receptor interaction correlation analysis, the recent Spectral-SAR approach is employed to introduce the Quantum-SAR (QuaSAR) “wave” and “conversion factor” in terms of difference between inter-endpoint inter-molecular activities for a given set of compounds; this may account for inter-conversion (metabolization) of molecular (concentration) effects while indicating the structural (quantum) based influential/detrimental role on bio-/eco- effect in a causal manner rather than by simple inspection of measured values; the introduced QuaSAR method is then illustrated for a study of the activity of a series of flavonoids on breast cancer resistance protein.

## 1. Introduction

Being used in Chemistry during the second half of 20th century as an extended statistical analysis [1–8], the quantitative structure-activity relationship (QSAR) method had attained in recent years a special status, officially certified by European Union as the main computational tool (within the so called “*in silico*” approach) for the regulatory assessments of chemicals by means of non-testing methods [9–15].

However, while QSAR primarily uses the multiple regression analysis [6–8], alternative approaches as such neuronal-network (NN) or genetic algorithms (GA) have been advanced to somehow generalize the QSAR performance in delivering a classification of variables used, in the sense of principal component analysis (PCA) and partial least squares (PLS) methodologies; still, the claimed advantage of the NN over QSAR techniques is limited by the fact the grounding physical-mathematical philosophies are different since highly non-linear with basic multi-linear pictures are compared, respectively [16–23].

Actually, the chemical-physical advantage of QSAR stands in its multi-linearity correlation that resembles with superposition principle of quantum mechanics, which allow meaningful interpretation of the structural (inherently quantum) causes associated with the latent or unobserved variables (sometimes called as *common factors*) into the observed effects (activity) usually measured in terms of 50%-effect concentration (EC_50_), associated with various types of bioaccumulation and toxicity [24].

Nevertheless, many efforts have been focused on applying QSAR methods to non-linearity features from where the “expert systems” emerged as formalized computer-based environments, involving knowledge-based, rule-based or hybrid automata able to provide rational predictions about properties of biological activity of chemicals or of their fragments; it results in various QSAR based databases: the model database (QMDB) - inventorying the robust summaries of QSARs that can be appealed by envisaged endpoint or chemical, the prediction database (QPDB) - when data from QMDB are used for further prediction to be stored, or together towering the chemical category database (CCD) documentation [25–31].

Therefore, although undoubtedly useful, the “official” trend in employing QSAR methods is to classify, over-classify and validate through (external or molecular test set) prediction, a gap between the molecular computed orderings and the associate mechanistic role in bio-/eco- activity assessment remains as large as the QSAR strategy has not turned into a versatile tool in identifying the inter-molecular role in receptor binding sites through recorded activities by means of structurally selected common variables; that is to use QSAR information for internal mechanistic predictions among training molecules to see their inter-relation respecting the whole class of observed activities employed for a specific correlation. Such an approach will also be helpful for checking the chemical domain spanned by training molecules – a feature of the paramount importance also for further external tests.

The present communication wishes to start filling this gap by deepening the modeling of inter-molecular activity through extending the main concepts of recent developed Spectral-SAR [32–40], developed the fully algebraic version of traditional statistically optimized QSAR picture, targeting the quantification of the competition between molecular inter-activity and inter-endpoints records.

## 2. QuaSAR Methodology

Paradoxically, the main problem for QSAR resides not in performing the correlation itself but setting the variable selection for it; the mathematical counterpart for such problem is known as the “factor indeterminacy” [41–45] and affirms that the same degree of correlation may be reached with in principle an infinity of latent variable combinations. Fortunately, in chemical-physics there are a limited (although many enough) indicators to be considered with a clear-cut meaning in molecular structure that allows for rationale of reactivity and bindings [46,47]. However, the main point is that given a set of *N*-molecules, one can chose to correlate their observed activities $A_{i=\bar{1,N}}$ with *M*-selected structural indicators in as many combinations as:

(1a) $$C=\sum_{k=1}^{M}C_{M}^{k},\;C_{M}^{k}=\frac{M!}{k!\left(M-k\right)!},$$

inked by different endpoint paths, as many as:

(1b) $$K=\Pi_{k=1}^{M}C_{M}^{k}$$

ndexing the numbers of paths built from connected distinct models with orders (dimension of correlation) from *k=*1 to *k=M.*

Basically, for each of the *C*-combinations a correlation (endpoint) QSAR equation is determined, say $Y_{l=\bar{1,C}}={\left\{y_{il}\right\}}_{\underset{l=\bar{1,C}}{i=\bar{1,N}}}$, containing all computed activities for all considered *N*-molecules within the *l*- selected correlation. Now, the Spectral-SAR version of QSAR analysis computes these activities in a complete non-statistical way, i.e. by assuming the vectors for both observed (activities) and unobserved (latent variables) quantities while furnishing their correlation throughout a specific S-SAR determinant obtained from the transformation matrix between the orthogonal (desirable) and oblique (input) correlations. Yet, besides producing essentially the same results as the statistical least-square fit of residues the S-SAR method introduces new concepts as:

- *endpoint spectral norm*
  (2) $$\left|\left||Y_{l}\rangle \right|\right|=\sqrt{\langle Y_{l}|Y_{l}\rangle }=\sqrt{\sum_{i=1}^{N}y_{il}^{2},}\;l=\bar{1,C}$$

allowing the possibility of the unique assignment of a number to a specific type of correlation, i.e. performing a sort of resumed quantification of the models [34];

- *algebraic correlation factor*
  (3) $$R_{ALG,l}=\frac{\left\||Y_{l}\rangle \right\|}{\left\||A\rangle \right\|}=\sqrt{\frac{\sum_{i=1}^{N}y_{il}^{2}}{\sum_{i=1}^{N}A_{i}^{2}},}l=\bar{1,C}$$

viewed as the ratio of the spectral norm of the predicted activity to that of the measured one, giving the measure of the overall (or summed up) potency of the computed activities respecting the observed one rather than the local (individual) molecular distribution of activities around the mean statistical yields; thus, it is a specific measure of the molecular selection under study, always with a superior value to that yielded from statistical approach [37], however preserving the same hierarchy in a shrink (less dispersive) manner being therefore better suited for intra-training set molecular analysis;

- *spectral path*, with the distance defined in the Euclidian sense as:
  (4) $$\left[l,l^{\prime }\right]=\sqrt{{\left(\left\||Y_{l}\rangle \right\|-\left\||Y_{l^{\prime }}\rangle \right\|\right)}^{2}+{\left(R_{l}-R_{l^{\prime }}\right)}^{2}},\forall \left(l,l^{\prime }\right)=\bar{1,C}$$

allows for defining complex information as path distances in norm-correlation space with norms computed from Equation (2) while correlation free to be considered either from statistical (local) or algebraically (global) – Equation (3) approaches; note that as far as computed activity *Y_l_* corresponds to the measured activity *A_l_* defined as logarithm of inverse of 50%-effect concentration (EC50), see bellow, both modulus of *Y_l_* vectors and *R* values have no units so assuring the consistency of the Equation (4).

- *least spectral path principle*, formally shaped as:
  (5) $$\delta \left[l_{1},\ldots l_{k}\ldots ,l_{M}\right]=0;\;l_{1},\ldots ,l_{k},\ldots ,l_{M}\;:ENDPOINTS$$

provides a practical tool in deciding the dominant {α,...}hierarchies along the paths constructed by linking all possible *k*-models (i.e. models with *k* correlation factors) from (1a) combinations selected one time each on a formed path – generating the so called “*M*-endpoints containing ergodic path on *K-*paths assembly” of (1b). However, the implementation of the principle (5) is recursively performed through selecting the least distance computed upon systematically application of Equation (4) on ergodic paths; if, by instance, two paths are equal there is selected that one containing the first two models with shorter norm difference in accordance with the natural least action; the procedure is repeated until all *C*-models where connected on shortest paths; there was already conjectured that only the first *M*-shortest paths (called as *α*_1_*,..., α_M_)* are enough to be considered for a comprehensive (and self-consistent) mechanistic analysis [34–40].

Nevertheless, for present purpose another two quantities are here introduced, namely:

▪ *inter-endpoint norm difference (IEND),*
  (6) $$\Delta Y_{l|l^{\prime }}=\left\||Y_{l^{\prime }}\rangle \right\|-\left\||Y_{l}\rangle \right\|,\;(l,l^{\prime })\in \;\left\{\alpha_{1},\ldots ,\alpha_{M}\right\}$$

that accounts for norm differences of the models lying on the *M*-shortest spectral paths linking *M*-from the *C*-models of Equation (1a);

▪ *inter-endpoint molecular activity difference (IEMAD),*
  (7) $$\Delta A_{i|j}^{l|l^{\prime }}=A_{j}^{l'}-A_{i}^{l}=\text{ln}\frac{1}{{\left(EC_{50}\right)}_{j}^{l^{\prime }}}-\text{ln}\frac{1}{{\left(EC_{50}\right)}_{i}^{l}}=\text{ln}\frac{{\left(EC_{50}\right)}_{i}^{l}}{{\left(EC_{50}\right)}_{j}^{l^{\prime }}}$$

is considered from activity difference between the fittest molecules (*i, j*), in the sense of minimum residues, for the models (*l, l’*) belonging to the shortest paths *α*_1_,..., *α_M_* for which the inter-endpoint norm difference is given by Equation (6).

This way, we can interpret the two fittest molecules (*i, j*) as reciprocally activated by the models (*l, l’*) through the spectral path whom they belong; put in analytical terms, the difference between quantities of Eqs. (6) and (7) may assure the “jump” or *transition activity* that turns the effect of *i* molecule on that of *j* molecule across the least spectral (here revealed as metabolization) path connecting the models *l* and *l’*:

(8) $$\text{ln}\frac{1}{q_{i|j}^{l|l^{\prime }}}\equiv \Delta Y_{l|l^{\prime }}-\Delta A_{i|j}^{l|l^{\prime }}\cdot$$

Note that if we rearrange Equation (8) in terms of 50-effect concentrations of Equation (7) one gets the wave-like form of molecular EC_50_ inter-molecular transformation:

(9) $${\left(EC_{50}\right)}_{i}^{l}={\left(EC_{50}\right)}_{j}^{l^{\prime }}q_{i|j}^{l|l^{\prime }}\text{exp}\left(i\Delta Y_{l|l^{\prime }}\right)$$

providing the analytic continuation in the complex plane for the *IEND* of Equation (6) was assumed, i.e. Δ*Y_l|l′_* → *i*Δ*Y_l|l′_* outside the factor $q_{i|j}^{l|l^{\prime }}$. Remark that although the differences in Eqs. (6) and (7) were consider mathematically, along the “arrow” *i-to-j*, the “quantum transformation” from Equation (9) suggests that the bio-chemical-physical equivalence (metabolization) of the concentration effects evolves *from-j-to-i*, revealing a typical quantum behavior with the factor $q_{i|j}^{l|l^{\prime }}$ playing the propagator role as the quantum kernels in path integral formulation of quantum mechanics [48].

Equation (9) stands as the present “quantum”-SAR equation since:

○ it involves *the wave-type* expression of molecular effect of concentration, however, for special selected molecules (the fittest out of the *C*-models) and for special selected paths (the least for the *M*-ergodic assembly), being *M* and *C* related by Equation (1a);
○ it provides the *specific transition* or specific transformation of the effect of a certain molecule into the effect of another special molecule out from the *N*-trained molecules, paralleling the phenomenology of consecrated quantum transitions;
○ it has the amplitude of transformation driven by the so called *quantum-SAR factor* of an exponential form
  (10) $$q_{i|j}^{l|l^{\prime }}=\text{exp}\left(\Delta A_{i|j}^{l|l'}-\Delta Y_{l|l^{\prime }}\right)$$
  defining the specific quantum-SAR wave;
○ it allows the *identity*
  (11) $${\left(EC_{50}\right)}_{i}^{l}={\left(EC_{50}\right)}_{i}^{l}$$
  when the reverse effects is considered
  (12) $${\left(EC_{50}\right)}_{i}^{l^{\prime }}={\left(EC_{50}\right)}_{i}^{l}\frac{1}{q_{i|j}^{l|l^{\prime }}}\text{exp}\left(-i\Delta Y_{l|l^{\prime }}\right)$$
  and substituted in the direct one (9), as absorption and emissions stand as reciprocal quantum effects;
○ it has a “phase” with unity norm, in the same manner as ordinary quantum wave functions, allowing the inter-molecular *“real” quantum-SAR transformation*
  (13) $$\left|{\left(EC_{50}\right)}_{i}^{l}\right|=q_{i|j}^{l|l^{\prime }}\cdot \left|{\left(EC_{50}\right)}_{j}^{l^{\prime }}\right|$$
  exclusively regulated by the quantum-SAR factor of Equation (10), in the same fashion as quantum tunneling is characterized by the transmission coefficient;
○ when *multiple transformations* take place across paths with multiple linked models, say (*l*, *l’*, *l’ ’*), the inter-molecular transformation *i→j→t* is characterized by the overall quantum-SAR factor (10) written as product of intermediary ones
  (14) $$q_{i|t}^{l|l^{\prime \prime }}=q_{i|j}^{l|l^{\prime }}\cdot q_{j|t}^{l^{\prime }|l^{\prime \prime }}$$
  due to the two-equivalent ways the ${\left(EC_{50}\right)}_{i}^{l}$ effect may be described directly from *t* or intermediated by *j* molecular effect transformations, respectively:
  (15) $$\begin{array}{l} \left|{\left(EC_{50}\right)}_{i}^{l}\right|=q_{i|t}^{l|l^{\prime \prime }}\cdot \left|{\left(EC_{50}\right)}_{t}^{l^{\prime \prime }}\right|\\ \;\;\;\;\;\;\;\;\;\;\;\;\;\;=q_{i|j}^{l|l^{\prime }}\cdot \left|{\left(EC_{50}\right)}_{j}^{l^{\prime }}\right|=q_{i|j}^{l|l^{\prime }}\cdot \left(q_{j|t}^{l^{\prime }|l^{\prime \prime }}\cdot \left|{\left(EC_{50}\right)}_{t}^{l^{\prime \prime }}\right|\right) \end{array}$$
  in the same way as the quantum propagators behave along quantum paths [48]; certainly, such contraction scheme may be generalized for least paths connecting the *M*-contained *k*-endpoints giving an overall quantum-SAR (“metabolization power”) factor as:
  (16) $$q_{i_{1}|i_{M}}^{l_{1}|l_{M}}=\prod_{w=2}^{M}q_{i_{w-1|i_{w}}}^{l_{w-1}|l_{w}}$$
○ Equation (9) supports the *self-transformation* as well, with the driven qua-SAR factor given by:
  (17) $$q_{i|j=i}^{l|l^{\prime }}=\text{exp}\left(-\Delta Y_{l|l^{\prime }}\right)$$
  during its evolution along the least paths when the same molecule (*i=j*) is metabolized by activating certain structural features (*l*≠*l’*) though specific indicators (variables) in correlation (bindings with receptor site); this case resembles the stationary quantum case according which even isolated (or with free motion), the molecular structures suffer dynamical wave-corpuscular or fluctuant transformation along their quantum paths;

With the present Qua-SAR methodology one can appropriately identify the molecular pairs that drive certain bio-/eco- activities against given receptor by means of selected descriptors in a “wave”-or “quantum” mechanistic formal way. The ultimate goal will be the computation of quantum-SAR factors along the least paths of actions that give the potential information of the conversion power of the fittest molecules in their specific bindings.

However, in order to practically understand the actual Qua-SAR approach all steps above will be in next specialized through an application for identifying the most involved polyphenolic molecules for their activity related to mammalian breast cancer.

## 3. Application to Flavonoids’ Anticancer Bioactivity

**Figure 1.**
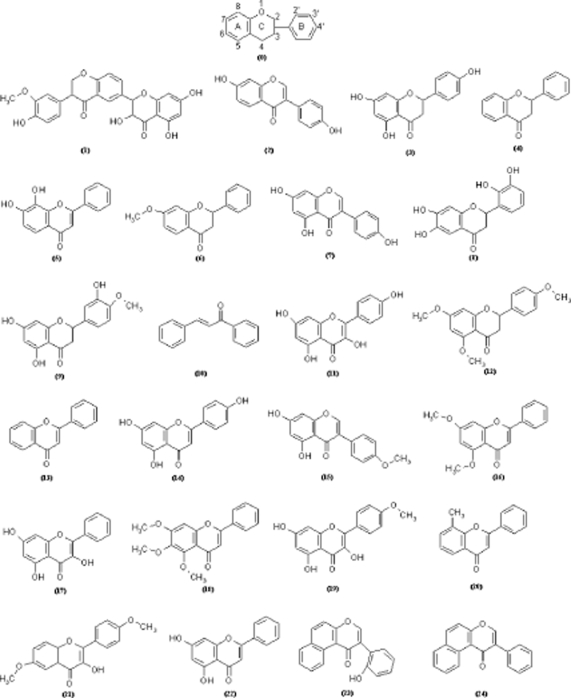
The studied flavonoids (with basic structure of as no.0 while the others are in the Table 1 characterized by associate QSAR data), covering the flavones, isoflavones, chalcones, flavonols and flavanones, as they assist the increase of mitoxantrone (MX) accumulation in BCRP-overexpressing MCF-7 MX100 breast cancer cells [51].

Although in general considered beneficial for their protective role in many age-related diseases - flavonoids (see Figure 1 – with the general scheme in no.0) should be more carefully studied since their pharmacokinetics are not entirely elucidated [49–54].

For instance, recently, it was inferred that for certain flavonoids such as chrysin, nbiochanin A and apigenin a very low micromolar concentration is capable of producing 50% (EC_50_) of the maximum increase in mitoxantrone (MX) inhibitor substrate accumulation (interaction) with breast cancer resistance protein (BCRP), helping in reversing the multidrug resistance (MDR) mechanism of overexpressing MCF-7 MX100 cancer cells [51–54].

Therefore, in order to assess the molecular role and structural- related mechanisms for potential lead compounds in the drug design for anti-cancer treatment, a series of representative classes of flavonoids have been employed, see Figure 1, with their recorded biological activities (A) among the computed transport (hydrophobicity-LogP), the electrostatic (polarizability POL), and steric (total energy at optimized 3D-configuration E_TOT_) Hantsch correlation variables [56], see Table 1, to successively provide the QSAR, S-SAR and finally to unfold the Qua-SAR analysis.

Note that in Table 1 the molecules were displayed in ascendant order of their recorded activities, from no. 1 to no. 24, for having present which is superior to which each time they are reciprocally quotation. Such an arrangement allows the construction of an activity differences chart, see Table 2, with great utility in establishing *the inter-endpoint molecular activity differences* of Equation (7) entering *quantum-SAR factor* of Equation (10).

Next, for computing the other influential activity difference in Qua-SAR, namely the *inter-endpoint norm difference* of Equation (6), the C = 10 possible endpoint models with data of Table 1 are in Table 3 presented. However, worth remarking that the traditional hydrophobicity factor LogP seems to have quite little or even no-influence from traditional statistical correlation (model Ia).

The first conclusion is that flavonoids have practically no exclusive or primarily role in drug transporting to BCRP site; still, the electrostatic influence through POL is practically missing as well (model Ib), while the stericity through E_TOT_ unfolds some statistically sensitive role in ligand (MX)-receptor (BCRP) binding (model Ic). The last assertion may also be sustained by going to the two-correlated parameters endpoint models, when one can see the confirmation of the stericity role through E_TOT_ correlation variable: while combination LogP∧POL does not improve the statistical correlation of model IIa significantly over single-parameter LogP∨POL correlations, the total energy presence provides better and better correlation behavior as it is combined with LogP (the model IIb) and with POL (the model IIc), respectively. Instead, when all the Hansch structural variables are taken into account the model III is generated with appreciable statistical correlation respecting the other computed combinations.

Overall, it cannot be inferred that LogP and POL does have no influence on correlation only because when alone they do not correlate at all with flavonoids’ bioactivity, because their cumulative presence in model III highly improves the single E_TOT_ correlation of model Ic as well as mixed correlations of bi-variable models IIb and IIc. Therefore, the mechanistic “alchemy” of structural features on molecular activity seems complex enough when all hydrophobicity, electrostatic and stericity influences combine as they are reciprocally activating one each other with a superior resultant in modeling ligand-receptor binding.

Yet, the algebraic correlation factors in Table 3 deserve special discussion: it is clear that as they are not measuring the dispersive character of the local computed (molecular) points against the average recorded activity as statistical metrics do, their values are all close to unity and close to each other as well; however, they are modeling another reality of computation, being closer to path integral approach than to differential analysis, through indexing the global behavior or the total length of the computed vector to the recorded one. Still, while between the algebraic and statistical correlations only an indirect connection exists [37], the one-to-one hierarchical ordering of models is always recorded thus supporting the usefulness of using algebraically scale when the shrink of correlation factors is more favorable. For instance, in the present case, as above revealed, according to the statistical analysis, there seems that LogP (Ia) and POL (Ib) have no influence on correlation, while when combined with E_TOT_ in model III they considerably enrich the single E_TOT_ correlation power of model Ic. Such behavior shows that orthogonal, i.e. independent, descriptors may provide better results when are combined than when considered apart due to the increase of the (inter) correlation space.

Having performed the QSAR analysis, the specific Spectral-SAR stage can be unfolded by means of the *(K* = 9, *M =* 3) ergodic paths with the spectral Euclidian lengths given by Equation (4) in both statistical and algebraic frameworks, as shown in Table 4. Next, the least *M* = 3 paths with the dominant *M*-factors influence are selected by applying the above exposed recursive rule of *least path principle* resumed by Equation (5). Remarkably, there follows that the resulting alpha (α), beta (β), and gamma (γ) most influential paths are identically shaped no matter whether statistical or algebraically schemes are undertaken. This result, although not necessarily viewed as a general rule, shows that in this specific case the algebraically analysis leaves with systematically the same mechanistically results as those obtained with statistical tools. However, once more, we stress on that algebraically measure may give more realistic inside in the Q(Spectral)-SAR phenomenology since its inner vectorial and norm-based algorithm accounting for each individual molecular contribution to the whole activity “basin” rather than respecting the average activity.

Going now to the individual molecular level analysis, Table 5 lists the residual activities between computed and observed activities for each of considered models, distributed along the already identified least paths. At this instance, the most fitted molecule is outlined out of each endpoint; most impressive, the actual research selected the same molecule as the best fitted one along the both α and β paths, namely molecule no. 12 (4′-5,7-trimethoxyflavanone) and molecule no. 13 (flavone), respectively. Moreover, these molecules are not among the most potent one respecting the observed activity of Table 1, being situated at the middle to second-half panel of the 24 molecules considered.

Such result tells us that the maximum recorded activity is not necessarily that one induced by *specific* chosen structural variables (here as LogP, POL, and E_TOT_). This is the case of the most fitted molecule on the most correlated endpoint (III) appeared to be no.3 (naringenin), with low activity on the observed range compared with the no. 25 (7,8-benzoflavone) in Table 1. Consequently, one can say that the first half of the observed activities in Table 1 may be attributed to certain physicochemical indicators with clear mechanistically roles, while the rest of observed activities may be due to other unidentified specific structural descriptors or even to non-specific ones (rooting in the sub-quantum nature of the particular observer-observed system). Nevertheless, this lower activity prescribed by the computational results is in accordance with the so called “homeopathic principle” prescribing cure by moderate-to-low active drugs while better monitoring their effects through controlled physico-chemical descriptors.

**Figure 2.**
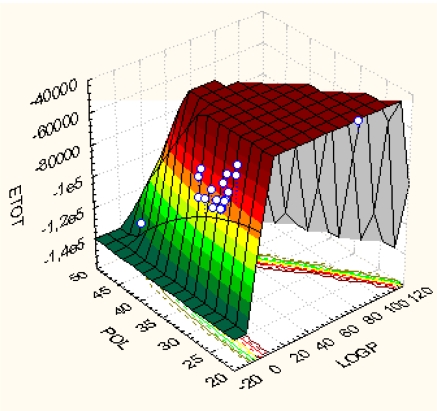
Quadratic 3D representation of LogP *vs.* POL *vs.* E_TOT_ variables’ fit employing the data of Table 1 [58].

For the sake of comparison, the actual Spectral(Qua)SAR results are to be compared with the consecrated Principal Component Analysis (PCA) [57]. This way, Figure 2 illustrates the graphical 3D correlations among the descriptors LogP, POL and E_TOT_ used in this study; it offers a visual way for assessing the almost no-correlation of LogP with other concerned variables, POL and E_TOT_, respectively. This lead with conclusion that LogP is almost orthogonal (independent) on (respecting) the other two Hantsch variables. Instead, when further performing the factor analysis, the Table 6 is obtained while clearly revealing the scarce correlation carried by considering LogP variable alone. This is in close agreement with the Spectral-SAR results, see above. In any case, the hydrophobicity description and its descriptor cannot be rejected only by factor analysis since it drives (firstly or latter) the inter-membrane interaction that is essential for drug-cell binding. Spectral- and Qua-SAR highly proved the important role hydrophobicity plays in combination with electrostatic (POL) and steric (E_TOT_) interactions. Moreover, while PCA shows the POL factor influence equals that of E_TOT_, whereas their role in correlation is sensible different in Spectral-SAR analysis (compare model Ib-last column of Table 3 with POL-last column of Table 6). However, again, this discrepancy is in the favor of S-SAR since the PCA results are due to the sensitive degree of POL-E_TOT_ correlation (see Figure 2), from where the PCA yield that POL and E_TOT_ display similar correlation power, while S-SAR includes also the orthogonalization of POL and E_TOT_ variables prior correlation takes effect and better discriminates among their influence in bonding.

Nevertheless, going ahead with the Spectral-SAR results the Qua-SAR factors may be immediately recover by employing the molecular activity differences from Table 2 for the best fitted molecules of Table 5 along the models of the most influential paths in molecular mechanism towards MX-BCRP binding. The resulted *IEND* and *IEMAD* of Table 7 are combined to produce the quantum-SAR factors of Equation (10) type for each two-molecules-two-models on specific paths, while the “metabolization power” *per* path is finally obtained by their couplings, according with multiplicative quantum rule of Equation (16). Worth noting that the overall quantum-SAR factors of paths are in total agreement with the previous spectral-SAR selected path hierarchy, i.e. the α path is associated with the highest q-SAR factor, being followed by that of β path and by that of γ one in last column of Table 7.

This result may be quite important if such a behavior may be proven to hold in general since it would allow the effective quantification of paths according with their metabolization power. However, such endeavor exceeds the present communication purpose and will remain as a future challenge in Qua-SAR studies.

**Figure 3.**
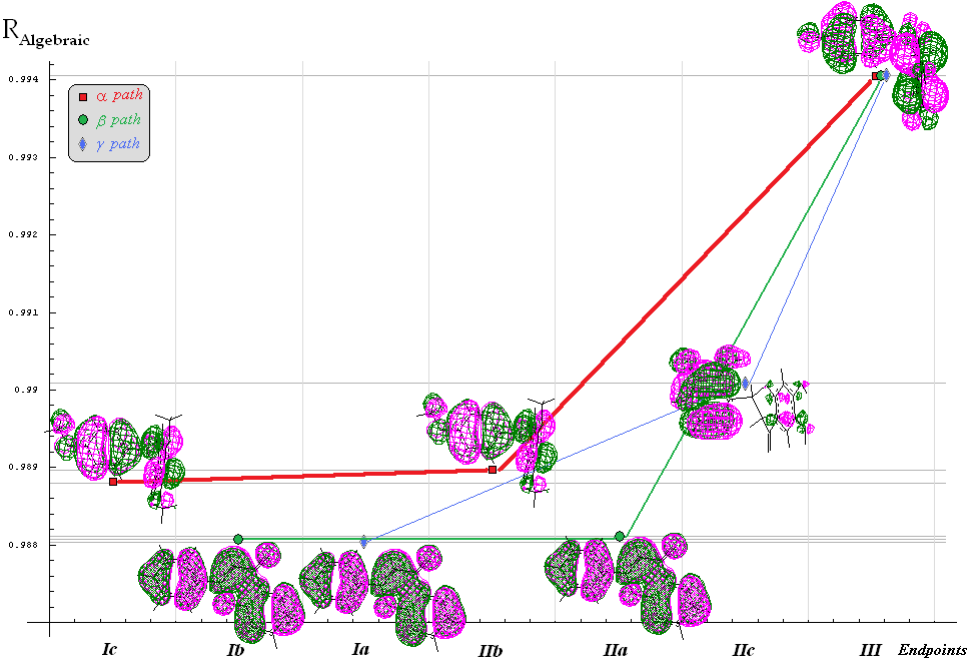
Spectral representation of the endpoints employed in designing the bioactivity mechanism for the molecules of Table 1, according with the algebraic correlation factors of Equation (3) in Table 3, across the shortest (three) paths identified from Table 4, while marking the fittest molecules’ orbital 3D-distribution for each considered model, i.e. molecule no. 12 (4′–5,7-trimethoxyflavanone) for the models Ic and IIb, molecule no. 13 (Flavone) for models Ib, Ia, and IIa, molecule no. 8 (6,2′,3′–7-tydroxyflavanone) for model IIc, and molecule no. 3 (naringenin) for model III, respectively.

Finally, all QSAR, Spectral-SAR and Qua-SAR computational results may be collected and resumed by associate “spectral” scheme for evolution of the fittest molecular structures along the endpoint models for the (*M*=)3 selected mechanistic paths of actions, see Figure 3. Note that algebraic correlation environment was chose as the “vertical” indicator for the degree with which a certain model reaches the observed activity in the vectorial norm sense (equally, the norm themselves could be used for ordinate axis [34]).

Going now to comment upon the “metabolization power” as indicated by the quantum-SAR factors on Figure 3, one can firstly observe that for the α path the “first movement” from the Ic (E_ToT_) to IIb (LogP∧E_TOT_) corresponds to quantum free motion so that the null IEMAD for molecule no. 12 (4′-5,7-trimethoxyflavanone) is carried; here, the quantum metabolization factor $q_{12|12}^{Ic|IIb;\alpha }$ is consumed only for strongly activating the membrane transporter feature (LogP) of the same molecule. Instead, on the last passage of the α path the factor $q_{12|13}^{IIb|III;\alpha }$ is responsible for converting the electrostatic (POL) influence of the flavonoids no. 12 towards no.3 (naringenin) activity as well as for reverse-O-methylation (methoxylation) of oxygens in positions 5 and 7 (on ring A) and 4′ (on ring B) respecting the molecular pattern no.0 in Figure 1, respectively. Such result is in fully accordance with the reverse quantum influence that is at the foreground of quantum-SAR factor conversion prescribed by Equation (9), i.e. quantifying the power of back transformation of molecular EC50s respecting the “arrows” of IEND and IEMAND in Equation (10). However, the fact that such transformation is the first one acting at molecular level is sustained also by optimized 3D configurations of involved molecules no. 12 and 3, being both with rings A and B spatially bent in Figure 3 respecting the ring C of the planar pattern no.0 of Figure 1.

A somewhat different situation is met for β path in Figure 3; in its first part a higher q-SAR factor ( $q_{13|13}^{Ib|IIa;\beta }>q_{12|12}^{Ic|IIb;\alpha }$) is needed for activating the transporter hydrophobicity feature in model IIa (LogP∧ POL) starting from model Ib (POL), while in its second part the molecule no. 13 (flavone) is shown to be metabolized in molecule no. 3 (naringenin) by a direct hydroxylation in positions 5, 7 (on ring A) and 4′ (on ring B), the same as before, respecting the molecular pattern no.0 in Figure 1, by a smaller q-SAR factor, $q_{13|3}^{IIa|III;\beta }<<q_{12|3}^{IIb|III;\alpha }$, compared with that involved in the previous alpha path. Despite these, the overall quantum factor of beta path is lower than that of alpha, meaning a decrease capacity of metabolization since direct addition is involved, contrarily to the ordinary “inverse” Quantum-SAR transformation of Equation (9), while stericity (here founded as the most influential QSAR variable) is triggered by more steric energy difference consumed between the planar optimized configuration of molecule no. 13 on that spatially bended of molecule no. 3 in Figure 3.

Even more metabolization “operations” take place along the γ path of Figure 3: there is started on the same planar configuration of molecule no.13 (flavone); then, the q-SAR factor $q_{13|8}^{Ia|IIc;\gamma }$ turn it into molecule no. 8 (6,2′,3′-7-hydroxyflavanone) by hydroxylation on the indicated positions (6 and 7 for ring A and 2′ and 3′ for ring B respecting the pattern molecule no.0 of Figure 1) while activating electrostatic and steric factors in model IIc (POL∧Etot) from independent hydrophobicity factor of model Ia (LogP) – a complex movement that explain why this molecular path comes at the final, with less probability and potency; nevertheless, this path has on its last passage no less complex transformation, i.e. turning the molecule no. 8 into no. 3 one by combined reverse hydroxylation in positions 2′ and 3′ with direct hydroxylation of position 4′ on B ring and with movement from ortho (6) – to – para (5) of hydroxyl group on ring A respecting pattern molecule no. 0 of Figure 1, respectively; the transformation efficiency $q_{8|3}^{IIc|III;\gamma }$ is a bit higher than on the first part of the path since it require less steric energy consumption to bent the ring C respecting the A-B ones while accounting for electronic delocalization density (orbitals) over them until the configuration of molecule no. 3 is reached in Figure 3.

Overall, it is clear that the Qua-SAR scheme offers a quantification recipe along the most effective spectral paths combined with most fitted molecules for a trial basin of analogues compounds and structural variables. In the present case there was revealed that the energetic steric factor E_ToT_ seems to mainly drive the mechanistic molecular transformation in MX-BCRP binding phenomenology, while the molecule no. 3 (naringenin) appears as the best fitted molecules belonging to the most relevant endpoint, in clear disjunction with the roughly molecular selection upon initial input observed activity data. That is, naringenin (no. 3) is shown to be the best adapted molecule for the actual LogP∧POL∧E_TOT_ structural (independent) factors being metabolized from molecules as 6,2′,3′-7-hydroxyflavanone (no. 8), 4′-5,7-trimethoxyflavanone (no. 12), and flavone (no.13) by specific molecular mechanistically paths. However, there appears that these molecules are not linked even through the paths with the most active compounds of Table 1; statistically, this can be explained by the so called “regression towards the mean” effects, in the sense that the best correlations translated to the compounds found in the middle of the mentioned sorted Table 1; from the structural point of view such behavior may attributed to the specific parameters used for correlations that best describe molecules with specific groups, most favorable for the descriptor’s nature.

On the other hand, the present study affirms the position 7 of ring A and position 4′ of ring B respecting the pattern molecule no. 0 of Figure 1 as the most suitable ones for producing an increase in BCRP inhibition activity, given that these positions belong to the α and β paths and being common to the rest of spectral paths as well. Instead, the position that does not appear at all in any of the α, β, or γ paths, namely position 8 on ring B may present adverse drug interactions.

Further Qua-SAR studies are necessary and will be developed for exploring other bio- and eco-active compounds for their interactions with organs and organisms; they may hopefully lead to a coherent analytical picture of chemical-biological bonding focused on selecting the most adapted molecules and of the most privileged molecular positions for delivering controlled structural based chemical reactivity and biological activity.

## 4. Conclusions

The modern *in silico* (computational) chemical analysis respecting the bio- activity and availability of analogues substances, potentially beneficial or detrimental for specific interaction in organs and organisms, faces with a paradoxical dichotomy: if searching for the best correlation useful for *prediction* of specific molecular bio- or eco- activity QSAR models involving un-interpretable many latent variables may be produced, while always remaining the question of correlation factor indeterminacy (i.e. the assumed descriptors can be at any time replaced with other producing at least the same correlation performances); instead, when restricting the analysis to search for molecular design and mechanisms throughout performing SARs by means of special structural indicators for a given class of relevant molecules, arises the price of limiting the use of generated models for further prediction. The present communication is mainly devoted in developing the second (Q)SAR facet by extending the recent introduced notion of spectral-path-linking-endpoints and the associate least action principle to spectral path quantification, in terms of the best fitted molecules, along the contained computed models, by means of the introduced q(uantum)-SAR factor within the generally called Quantum-SAR (QuaSAR) methodology.

As an application, for representative flavonoids’ inhibiting activities on breast cancer resistant protein there was clearly shown that the newly introduced q-SAR factor offers relevant analytical characterization of previously conceptually introduced spectral path hierarchy; moreover, the present QuaSAR may allow interpretation inter-conversion of concerned molecules’ towards receptor binding since belonging to the same class of analogs, while they certainly undertaking such transformation during their interaction with macromolecules, proteins and enzymes present on cellular walls or with *in vivo* environment.

Basically, the QuaSAR stands as the first step in assessing the quantum mechanically equivalent of wave function to the sample of molecules interacting with a specific organism site; it will eventually lead with the hyper-wave function with the help of which the associate hyper-density probability of binding (metabolization) is to be computed; the last information may provide the density probability map of the ligand-receptor interaction abstracted from the structural Spectral-Qua-SAR correlations; with this tool the molecular design of new chemical structures may be appropriately undertaken.

However, the actual QuaSAR scheme and quantum factor carry the main features of quantum dynamical systems and may stimulate future computational and conceptual developments in molecular design for structurally controlled activity. Further generalization of the present QuaSAR method to modeling all potential inter-conversions of employed molecules involved in correlation as well as for establishing their quantum metabolization complete map (through, for instance, hydrophobic, electrostatic and steric barrier tunneling) is actually in progress and will be reported in subsequent communications.

## Figures and Tables

**Table 1.** The flavonoids of Figure 1 arranged by their ascending observed activities, defined as A= -log_10_(EC_50_[μM]) [51], along the associate computed structural parameters like the hydrophobicity (LogP), electronic cloud polarizability (POL) and the ground state configurationally optimized total energy (E_TOT_) [55].

**No.**	**Molecular Name**	**Activity**	**Structural parameters**		
		**A**	**LogP**	**POL(Å^3^)**	**E_TOT_(kcal/mol)**
**(1)**	Silybin	3.74	2.03	45.68	− 146625.1875
**(2)**	Daidzein	4.24	1.78	26.63	− 76984.7109
**(3)**	Naringenin	4.49	1.99	27.46	− 85032.9218
**(4)**	Flavanone	4.6	2.84	25.55	− 62849.3125
**(5)**	7,8-Dihydroxyflavone	4.7	1.75	26.63	− 76982.1328
**(6)**	7–Methoxyflavanone	4.79	2.59	28.02	− 73823.8046
**(7)**	Genistein	4.83	1.50	27.27	− 84380.7578
**(8)**	6,2′,3′-7-Hydroxyflavanone	4.85	1.70	28.10	− 92422.6640
**(9)**	Hesperetin	4.91	1.73	29.93	− 96003.9921
**(10)**	Chalcone	4.93	3.68	25.49	− 55450.1093
**(11)**	Kaempferol	5.22	0.56	27.90	− 91770.5859
**(12)**	4′-5,7-Trimethoxyflavanone	5.25	2.08	32.96	− 95768.9062
**(13)**	Flavone	5.4	2.32	25.36	− 62196.3437
**(14)**	Apigenin	5.78	1.46	27.27	− 84379.8593
**(15)**	Biochanin A	5.79	1.53	29.10	− 87961.2812
**(16)**	5,7-Dimethoxyflavone	5.85	1.81	30.30	− 84139.4687
**(17)**	Galangin	5.92	0.85	27.27	− 84376.8359
**(18)**	5,6,7–Trimethoxyflavone	5.96	1.56	32.77	− 94976.1875
**(19)**	Kaempferide	5.99	0.60	29.74	− 95351.3984
**(20)**	8-Methylflavone	6.21	2.79	27.19	− 65789.9218
**(21)**	6,4′–Dimethoxy-3-hydroxy-flavone	6.35	0.41	31.13	− 92162.7187
**(22)**	Chrysin	6.41	1.75	26.63	− 76986.1171
**(23)**	2′-Hydroxy-α-naphtoflavone	7.03	3.07	33.26	− 82027.8359
**(24)**	7,8 – Benzoflavone	7.14	3.35	32.63	− 74634.5234

**Table 2.** The anti-symmetric matrix of the inter-molecular activity differences for the working flavonoids of Table 1.

**1**	**2**	**3**	**4**	**5**	**6**	**7**	**8**	**9**	**10**	**11**	**12**	**13**	**14**	**15**	**16**	**17**	**18**	**19**	**20**	**21**	**22**	**23**	**24**	
0	0.5	0.75	0.86	0.96	1.05	1.09	1.11	1.17	1.19	1.48	1.51	1.66	2.04	2.05	2.11	2.18	2.22	2.25	2.47	2.61	2.67	3.29	3.4	**1**
	0	0.25	0.36	0.46	0.55	0.59	0.61	0.67	0.69	0.98	1.01	1.16	1.54	1.55	1.61	1.68	1.72	1.75	1.97	2.11	2.17	2.79	2.9	**2**
		0	0.11	0.21	0.3	0.34	0.36	0.42	0.44	0.73	0.76	0.91	1.29	1.3	1.36	1.43	1.47	1.5	1.72	1.86	1.92	2.54	2.65	**3**
			0	0.1	0.19	0.23	0.25	0.31	0.33	0.62	0.65	0.8	1.18	1.19	1.25	1.32	1.36	1.39	1.61	1.75	1.81	2.43	2.54	**4**
				0	0.09	0.13	0.15	0.21	0.23	0.52	0.55	0.7	1.08	1.09	1.15	1.22	1.26	1.29	1.51	1.65	1.71	2.33	2.44	**5**
					0	0.04	0.06	0.12	0.14	0.43	0.46	0.61	0.99	1	1.06	1.13	1.17	1.2	1.42	1.56	1.62	2.24	2.35	**6**
						0	0.02	0.08	0.1	0.39	0.42	0.57	0.95	0.96	1.02	1.09	1.13	1.16	1.38	1.52	1.58	2.2	2.31	**7**
							0	0.06	0.08	0.37	0.4	0.55	0.93	0.94	1	1.07	1.11	1.14	1.36	1.5	1.56	2.18	2.29	**8**
								0	0.02	0.31	0.34	0.49	0.87	0.88	0.94	1.01	1.05	1.08	1.3	1.44	1.5	2.12	2.23	**9**
									0	0.29	0.32	0.47	0.85	0.86	0.92	0.99	1.03	1.06	1.28	1.42	1.48	2.1	2.21	**10**
										0	0.03	0.18	0.56	0.57	0.63	0.7	0.74	0.77	0.99	1.13	1.19	1.81	1.92	**11**
											0	0.15	0.53	0.54	0.6	0.67	0.71	0.74	0.96	1.1	1.16	1.78	1.89	**12**
												0	0.38	0.39	0.45	0.52	0.56	0.59	0.81	0.95	1.01	1.63	1.74	**13**
													0	0.01	0.07	0.14	0.18	0.21	0.43	0.57	0.63	1.25	1.36	**14**
														0	0.06	0.13	0.17	0.2	0.42	0.56	0.62	1.24	1.35	**15**
															0	0.07	0.11	0.14	0.36	0.5	0.56	1.18	1.29	**16**
																0	0.04	0.07	0.29	0.43	0.49	1.11	1.22	**17**
																	0	0.03	0.25	0.39	0.45	1.07	1.18	**18**
																		0	0.22	0.36	0.42	1.04	1.15	**19**
																			0	0.14	0.2	0.82	0.93	**20**
																				0	0.06	0.68	0.79	**21**
																					0	0.62	0.73	**22**
																						0	0.11	**23**
																							0	**24**

**Table 3.** QSAR equations through Spectral-SAR multi-linear procedure [32–34] for all possible correlation models considered from data of Table 1; here |*X*_0_ 〉 is the unitary vector|11...1_24_〉, while the structural variables are set as |*X*_1_〉 = *LogP*, |*X*_2_〉 = *POL,* and |*X*_3_〉 = *E_TOT_*; the predicted activities’ norms where calculated with Equation (2), while the algebraic correlation factor of Equation (3) uses the measured activity of ‖| *A*〉‖ = 26.9357 computed upon Equation (2) with data of Table 1; R_Statistic_ is the traditional Pearson correlation factor [1–8].

**Model**	**Variables**	**(Q/S-)SAR Equation**	‖|*Y*〉 *^PREDICTED^*‖	R_Algebraic_	R_Statistic_
***Ia***	|*X*_0_>, |*X*_1_>	|*Y*>^Ia^ = 5.39837|*X*_0_>+0.0179106|*X*_1_>	26.6138	0.988049	0.0175601
***Ib***	|*X*_0_>, |*X*_2_>	*|Y*>^Ib^ = 5.67735 *X*_0_>–0.00834411*|X*_2_*>*	26.61425	0.988065	0.0409922
***Ic***	*|X*_0_*>, |X*_3_*>*	*|Y*>^Ic^ = 6.48303*|X*_0_>+0.0000124625*|X_3_*>	26.6344	0.988812	0.252513
***IIa***	*|X*_0_*>, |X*_1_〉*,|X*_2_*>*	*|Y*>^IIa^ = 5.64318*|X*_0_> +0.0178242 |*X*_1_〉–0.00833676|*X*_2_>	26.614349	0.988069	0.0445618
***IIb***	*|X*_0_>, *|X*_1_>,*|X*_3_>	*|Y*>^IIb^ = 6.93331*|X*_0_*>* − 0.120924*|X*_1_>+0.0000150708*|X*_3_>	26.638	0.988947	0.273909
***IIc***	*|X*_0_>, *|X*_2_>,*|X*_3_>	*|Y*>^IIc^ = 4.99884*|X*_0_> +0.122989*|X*_2_>+0.0000376701 *|X*_3_*>*	26.6681	0.990063	0.409837
***III***	*|X*_0_>, *|X*_1_>,*|X*_2_>, *|X*_3_>	*|Y*>^III^ = 5.59424*|X*_0_>–1.05993|*X*_1_>+0.400704*|X*_2_*>*+0.000117452*|X*_3_>	26.7758	0.994064	0.708509

**Table 4.**
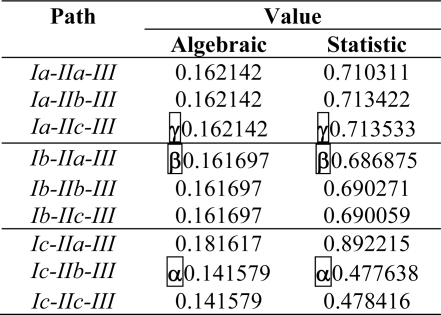
Synopsis of paths connecting the endpoints of Table 3 in the norm-correlation spectral-space.

**Table 5.**
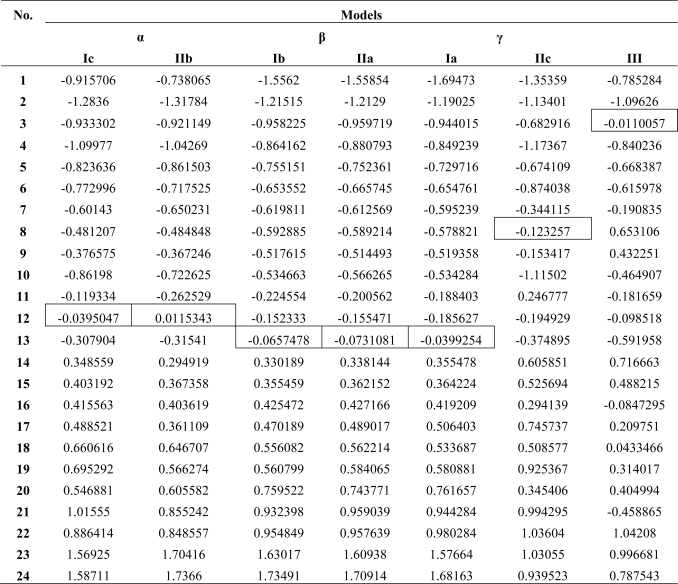
Residual activities A_i_ – Y_i_^Model^ of the compounds of Table 1 for the Spectral-SAR models of Table 3 ordered according with the alpha, beta and gamma paths of Table 4; that residue which is closes to zero in each considered endpoint is marked by a line border.

**Table 6.** Principal Component Analysis (PCA) for the data of Table 1 within unrotated (unnormalized) factor score coefficients [58].

	**PC1**	**PC2**	**PC3**	**Multiple**
*Eigenvalue:*	1.958158	0.892127	0.149715	**PC1–PC3**
*% total variance:*	65.27195	29.73757	4.99049	**factors’**
	
***Variable***	***Factors’ coefficients***			**R^2^**
LogP	0.232179	−0.997780	0.20467	0.083712
POL	−0.472177	−0.302902	−1.79349	0.716820
E_TOT_	0.483556	0.183309	−1.84956	0.728872

**Table 7.** Determination of the quantum-SAR, see Equation (10) with Eqs. (6) and (7), associate with certain couple of molecules involved in activating specific structural quantum indices (or their combinations) driving spectral paths of Table 4, by employing minimum residue recipe throughout Table 5 for each considered endpoint, as well as the associate recorded bioactivity differences of Table 2, respectively.

Path	$\Delta Y_{l|l^{\prime }}^{PATH}$ (IEND)#	$\Delta A_{i|j}^{l|l^{\prime }}$ (IEMAD)^♣^	$q_{i|j}^{l|l^{\prime };PATH*}$*	*q^PATH^*
α	$\Delta Y_{Ic|IIb}^{\alpha }=0.00364573$	$A_{12|12}^{Ic|IIb}=0$	$q_{12|12}^{Ic|IIb;\alpha }=0.991641$	*q*^α^ =0.125464
	$\Delta Y_{IIb|III}^{\alpha }=0.137836$	$A_{12|3}^{IIb|III}=-0.76$	$q_{12|3}^{IIb|III;\alpha }=0.126521$	
β	$\Delta Y_{Ib|IIa}^{\beta }=0.0000989324$	$A_{13|13}^{Ib|IIa}=0$	$q_{13|13}^{Ib|IIa;\beta }=0.999772$	*q*^β^=0.0848036
	$\Delta Y_{IIa|III}^{\beta }=0.161487$	$A_{13|3}^{IIa|III}=-0.91$	$q_{13|3}^{IIa|III;\beta }=0.0848229$	
γ	$\Delta Y_{Ia|IIc}^{\gamma }=0.0542592$	$A_{13|8}^{Ia|IIc}=-0.55$	$q_{13|8}^{Ia|IIc;\gamma }=0.248737$	*q*^γ^ =0.0847168
	$\Delta Y_{IIc|III}^{\gamma }=0.107771$	$A_{8|3}^{IIc|III}=-0.36$	$q_{8|3}^{IIc|III;\gamma }=0.340588$	

^#^ Inter-Endpoint Norm Difference, Equation (6);

♣ Inter-Endpoint Molecular Activity Difference, Equation (7);

* Note that here the basic relation of Equation (10) was considered in decimal base since originally, the associated activities in Table 1 were as such defined.
